# Pre-linguistic Vocal Trajectories at 6–18 Months of Age As Early Markers of Autism

**DOI:** 10.3389/fpsyg.2016.01595

**Published:** 2016-10-19

**Authors:** Natasha Chericoni, Daniele de Brito Wanderley, Valeria Costanzo, Andréa Diniz-Gonçalves, Marluce Leitgel Gille, Erika Parlato, David Cohen, Fabio Apicella, Sara Calderoni, Filippo Muratori

**Affiliations:** ^1^IRCCS Stella Maris FoundationPisa, Italy; ^2^Nucleo Interdisciplinar de Intervenção Precoce da BahiaBahia, Brazil; ^3^Service de Pédopsychiatrie- Hôpital Necker Enfants MaladesParis, France; ^4^School of Medicine, Federal University of Minas GeraisBelo Horizonte, Brazil; ^5^Department of Child and Adolescent Psychiatry, Groupe Hospitalier Pitié-Salpérière, Assistance Publique - Hôpitaux de ParisParis, France; ^6^Institut des Systèmes Intelligents et de Robotiques, Université Pierre et Marie CurieParis, France; ^7^Stella Maris Mediterraneo FoundationChiaromonte, Italy; ^8^Department of Clinical and Experimental Medicine, University of PisaPisa, Italy

**Keywords:** autism spectrum disorder, early language development, vocalizations, babbling

## Abstract

This study explores pre-linguistic vocal trajectories in infants with autism spectrum disorder (ASD) during caregiver-infant interaction. Home videos were obtained from 10 infants with ASD and 10 typically developing infants (TD), covering three time periods: 0–6 months (T1, 47 video sequences), 6–12 months (T2, 47 video sequences), and 12–18 months (T3, 48 video sequences). In total 142 video sequences were analyzed. Vocalizations, long reduplicated babbling, 2-syllable babbling, and first words were investigated longitudinally. Face-gazing was also analyzed, to evaluate the social quality of vocal behaviors. Results show a lower rate of vocalizations in the ASD group at T2, and a lower rate of first words at T3, compared to the TD group. However, the prevalence of non-social babbling, appeared higher in the ASD group. The implications of these findings for screening programs are discussed.

## Introduction

Autism Spectrum Disorder (ASD) is defined as a neurodevelopmental disorder that interferes with social interaction and communication, and impacts a person's interests which become repetitive and restricted (American Psychiatric Association, 2013). Early detection of ASD is a prerequisite for early intervention which in turn may mitigate the severity of core and associated features of autism (Warren et al., 2011), and improve the long-term outcome of treated patients (Estes et al., 2015). Unfortunately, early detection is rare and ASD is typically diagnosed relatively late, after 3 years of age (Mandell et al., 2010; Valicenti-McDermott et al., 2012). More research is needed to study the early onset and development of ASD, using both retrospective and prospective methods. The identification of developmental trajectories specific to ASD is in fact pivotal for the creation of early screening tools, which could improve early detection and timely treatment. Indeed, it is not simple to develop screening instruments which address early infancy because autism rarely displays a clear and rigid pattern during the first year of life whereas it does in later years (Saint-Georges et al., 2010). For example, Gabrielsen et al. (2015) reported that young children who had autism showed more typical behavior (89% of the time) than atypical behavior: They could gaze at people's faces, turn toward voices, respond to their name when called, and express interest in communication. Searching for early signs, much attention has been devoted to the lack of gaze-following and joint attention (Dawson et al., 2004; Naber et al., 2007; Clifford and Dissanayake, 2008), and more recently to other non-social symptoms such as motor development (Guinchat et al., 2012; Leonard et al., 2014). Delays in posture development and in fine motor skills have been found in infants at high risk for ASD as early as 6 months (Iverson and Wozniak, 2007; Bhat et al., 2012; Nickel et al., 2013; Libertus et al., 2014). Some researchers believe that, as opposed to being unrelated to the social-communicative domain, they may indeed be connected to later language impairments. Nickel et al. (2013), for example, hypothesize that a slower development in sitting and standing postures may exert negative cascading effects also in the communicative domain. This hypothesis appears to be supported by research into typical development, where the emergence of sitting skills has been correlated to receptive vocabulary size at 10 and 14 months of age (Libertus and Violi, 2016).

Vocalization and babbling, which can be considered the earliest expression of language development, have so far received poor attention as early markers of autism. This is surprising for at least three reasons: (1) parents' first concerns and request for medical consultation are often related to their child's language delay (Coonrod and Stone, 2004) (2) autistic children's expressive and receptive language presents unique characteristics that distinguish communication impairments in ASD from other developmental disorders, e.g., unusual tone, echolalia, repeated humming (Tager-Flusberg and Caronna, 2007; Demouy et al., 2011) (3) treatment response is often evaluated on the basis of language gains (Spreckley and Boyd, 2009; Eapen et al., 2013).

The pre-linguistic vocal pattern profile in autistic children still remains uncertain; most studies have focused on verbal development between the first and the second birthdays which appears slower or decreasing, compared to both TD children and children with global or specific language delay (Landa and Garrett-Mayer, 2006; Barbaro and Dissanayake, 2012). To provide a more in depth understanding of early vocal trajectory in children with ASD, some researches have used video segments of the child interacting with the examiner during the MSEL (Ozonoff et al., 2010) or the CSBS-DP (Shumway and Wetherby, 2009; Landa et al., 2013; Plumb and Wetherby, 2013); other researches have analyzed children's vocal or gesture behaviors during interaction with a caregiver using a standard set of toys during laboratory visits (Paul et al., 2011; Rozga et al., 2011) or have asked parents to provide home videotapes (Watson et al., 2013; Winder et al., 2013; Patten et al., 2014). Results from these studies show that in the second year of life children with ASD communicate at a significantly lower rate compared to TD children (Winder et al., 2013; Patten et al., 2014), showing a significantly lower proportion of vocalizations with consonants or with speech sounds and a significantly higher proportion of atypical vocalizations (Plumb and Wetherby, 2013). Gabrielsen et al. (2015) have demonstrated that atypical percentage scores in vocalization showed the highest correlation to the three main ADOS scores. These characteristics are already evident at 12 months, when ASD infants produce fewer consonant types and canonical syllable shapes (Paul et al., 2011; Patten et al., 2014). Prior to 9 months however there is little literature on vocal patterns and no differences have been identified so far in comparison to TD children (Ozonoff et al., 2010; Paul et al., 2011; Rozga et al., 2011).

According to Iverson et al. (2007), reduplicated babbling, after vocalization, is the most important milestone in the first year of life of TD infants. Between 6 and 10 months, infants begin to produce simple syllables formed by a consonant and a vowel in a repetitive way [babababa]; these sequences join sensory and motors aspects of vocalizations. After this first period of reduplicated babbling, another type of babbling, characterized by just two syllables and with a lot of variations appears (baba; baga; gaba) and acts as a bridge toward first words (De Boysson-Bardies, 1996; Le Normand, 2007). Oller et al. (1999) found an association between late onset of babbling and a delay in the onset of speech production; they also suggest that late onset of babbling may be a predictor of disorders like autism. More recently, Iverson and Wozniak (2007), in a study on infants at risk because of an older sibling diagnosed with autism, found differences in babbling and first words that are both poorly organized and infrequent in infants who are developing autism. However, studies on the first year of life are still quite scarce, and more data from the first year of life should be collected in order to better understand the different developmental trajectory that characterizes vocal production in children with ASD.

In a previous study (Apicella et al., 2013) using home videos, we found that lower rates in the amount of vocalizations (any kind of utterance produced by infants) significantly distinguished ASD from TD during the 6–12 months age period. In particular, we found a decline in vocalizations used to respond in ASD and a significant growth in TD. In the present study we aimed to investigate pre-linguistic vocal trajectories in more detail, distinguishing between early vocalizations, babbling and first words, and chose to extend data analysis to the 12–18 months age period. Hence, we compared vocal trajectories during the first 18 months of life, in infants with ASD in comparison with TD infants. Apart from the frequency of different vocal behaviors, we also investigated the quality of these behaviors, in terms of the presence or absence of Face-gazing. To study how language develops within the interactive context, we selected scenes of caregiver-infant interaction and chose to analyze the coupling of vocal behaviors and eye-gaze. Indeed, typically developing 2-month-old babies look at their caregiver's eyes with focused interest and take part in “proto-conversations,” timing their looks, smiles, and coos to fit the rhythm of the mother's “baby talk,” so that the mother feels sure the baby is trying to “talk” with her (Trevarthen, 2002). In ASD, however, intersubjectivity appears disrupted (Muratori and Maestro, 2007; Muratori et al., 2011), so it is possible that vocalizations might develop following a different trajectory. By observing whether face-gazing is present during early vocal behaviors, we investigated the social-communicative valence of pre-language productions in ASD infants.

Hence, the purpose of our study was two-fold: (1) to test the hypothesis that early vocal patterns (vocalizations, babbling, first words) are able to differentiate ASD from TD children at a certain point of development; (2) to test the hypothesis that the social quality of vocal behavior (i.e., presence or absence of Face-gazing coordinated with vocal production) widens the differences between the two groups.

## Methods

### Participants

This study is based on a retrospective analysis of home videos taken during the first 18 months of the child's life. The video material was collected over the past 20 years at the IRCCS Stella Maris Foundation in Pisa, a tertiary care university hospital for Child and Adolescent Psychiatry, associated with the University of Pisa and the National Health Service. The clinical data was collected both for diagnostic and research purposes during the diagnostic process these children underwent at the Stella Maris Institute. The study was carried out in accordance with the recommendations of the IRCCS Stella Maris Foundation institutional review board, with written informed consent from all subjects. For the purpose of this study, we analyzed home videos from two groups of children. The first group was composed of 10 children (M/F: 9/1) with a diagnosis of ASD performed by a child psychiatrist according to the DSM-5 criteria (American Psychiatric Association, 2013) and confirmed by ADOS and ADI-R scores. At the time of the diagnosis, their ages ranged between 4 and 6 years. They were also administered Griffiths Mental Developmental Scales—ER in order to determine intellectual functioning: General quotient (GQ) ranged from 49 to 73, with a group mean of 58.72 (*SD*: 8.23). The second group was composed of 10 TD children (M/F: 8/2) who were recruited among children attending a local kindergarten; these children did not present abnormal medical or developmental conditions as confirmed by non-clinical scores using the Child Behavior CheckList (Achenbach and Rescorla, 2000).

### Video collection and editing procedures

Families were asked to provide any videotape recorded during their child's first 2 years of life. They were told that the material would be analyzed as part of a study investigating the earliest phases of child development and were asked not to select the material themselves. Copies of the videos were made and coded with an ID number to preserve confidentiality. Written informed consent was collected from participating families in accordance with the Declaration of Helsinki. The selection of the video clips was carried out by a research assistant blind to the children's diagnoses. She was given access to a database of videos that had already been classified by age range (0–6, 6–12, 12–18 months) for previous studies. In order to investigate changes taking place over development and to be able to compare and add findings to previous research (e.g., Muratori et al., 2011; Apicella et al., 2013), we maintained this division in three age periods: 0–6 months (hereinafter T1), 6–12 months (hereinafter T2), 12–18 months (hereinafter T3). The research assistant was instructed to select any sequence of infant-caregiver interaction occurring during different daily routines, i.e., play, meal, and bath time (scenes in which the infant was playing by himself, or interacting with unfamiliar adults were not taken into consideration). The length of each video clip was set at a minimum of 30 s, and a maximum of 2 min, to ensure a minimum amount of interaction with the caregiver, and the use of at least 2 different sequences for each time period, per child. All participants included in the current study had 9 min of video compilations, 3 min for each time period (Table 1). Before proceeding with the coding of videos, a *T*-test was performed to check that the material selected for each time period was comparable across groups in terms of segment length, number and type of situations, and when necessary adjustments were made. The final sample consisted of 180 min (142 video sequences in total), with an average of 60 min (47.3 sequences) per time period (Table 2). The different kinds of contexts (play/meal/bath time) were represented in the same proportions in both groups. Overall, they were distributed in the following way: Play time: 23.24% in ASD; 21.13% in TD; meal time 16.20% in ASD and 14.08% in TD; bath time 11.97% in ASD and 13.38 in TD (Table 3).

**Table 1:** **Means and standard deviations for minutes and number of video sequences for infants of both groups (ASD,TD), at the three time periods (T1,T2,T3)**.

**Child**	**T1**		**T2**		**T3**	
	**Minutes Mean *(SD)***	**Sequences Mean *(SD)***	**Minutes Mean *(SD)***	**Sequences Mean *(SD)***	**Minutes Mean *(SD)***	**Sequences Mean *(SD)***
ASD	2.96 (0.16)	2.3 (0.48)	2.99 (0.1)	2.5 (0.53)	3.02 (0.21)	2.5 (0.53)
TD	3.09 (0.14)	2.4 (0.52)	3.01 (0.09)	2.2 (0.42)	2.93 (0.2)	2.3 (0.48)

**Table 2:** **Total minutes and number of video sequences for each group (ASD, TD) at the three time periods (T1,T2,T3)**.

**Group**	**T1**		**T2**		**T3**	
	**Total minutes**	**Total sequences**	**Total minutes**	**Total sequences**	**Total minutes**	**Total sequences**
ASD	29.6	23	29.9	25	30.2	25
TD	30.9	24	30.1	22	29.3	23
Total	60.5	47	60	47	59.5	48

**Table 3:** **Percentage of each activity type for both groups (ASD,TD) at each time period (T1,T2,T3)**.

	**T1**		**T2**		**T3**	
	**ASD(%)**	**TD(%)**	**ASD(%)**	**TD(%)**	**ASD(%)**	**TD(%)**
Play	7.04	7.75	7.75	6.34	8.45	7.04
Meal	4.23	3.52	6.34	5.63	5.63	4.93
Bath	4.93	5.63	3.52	3.52	3.52	4.23

### Observational behavior grid and coding procedures

All infant vocal productions were identified, using 2 s of silence between successive productions as the criterion for completion of one vocalization and the initiation of another. Vocal productions were classified into four major categories: (1) Vocalizations, consisting in vocal productions characterized by the production of vowels or non-reduplicated consonants and vowels (e.g., “aaaaaahhh,” “gaaaaaaah”); non-speech sounds such as squeals, yells, growls, or grunts were not coded (an exception was made for fussy vocalizations); (2) Long reduplicated babbling, that is vocal productions characterized by long strings of reduplicated babbling consisting in 3 or more units (e.g., “babababa”); (3) 2-syllable babbling, that is vocal productions characterized by a clear reduplicated babbling consisting in two identical or variegated syllables (e.g., “baba”; “baga”); (4) First words, that is vocal productions that were either conventional Italian words or approximations of the latter (e.g., “babbo” for “daddy”); jargon and idiosyncratic words were not included.

In order to assess the social quality of vocal productions we specified whether the child was gazing toward the caregiver's face while producing the utterance. Face-gazing was coded as a YES/NO variable: Face-gazing was considered present and coded as “Yes Face-gazing” when it lasted more than 1 s: Extremely brief or fleeting glances, lasting less than 1 s were coded as “No Face-gazing.” One coder (DW) was a clinical expert in autism and in early intervention; the other coder (AD) was a psychologist specialized in child development. Both were blind to group membership and performed the coding using the Observer XT 10.0 (Noldus, 2010), a professional software for the collection, management, and analysis of observational data. Inter-rater reliability was calculated directly by the Noldus Observer software and the values of κ were ≥ 0.70 for all variables.

### Data analysis

Statistical analyses were performed using R Software, Version 2.12.2. Due to the different length of video sequences, the frequency of each behavior was converted, respectively, to a ratio number of behaviors per time (hereinafter rate per minute). As the majority of variables was not normally distributed (as evaluated with the Shapiro-Wilk Test) analyses were performed with the Mann- Whitney non-parametric test in order to detect “between-group” differences (separately for T1, T2, and T3). “Within-group” differences were investigated using Friedman Test. *Post-hoc* analysis with Wilcoxon signed-rank tests was conducted with a Bonferroni correction applied, resulting in a significance level set at *p* < 0.017. We also performed a multivariate analysis using a Generalized Mixed Model including all variables that reached normal distribution via Log transformation. This was the case only for vocalization and Face-gazing with the Log (Variable) + 0.5. R package ≪ lme4 ≫ was used, and *p*-values were computed using a normal approximation. Repeated measures were modeled with a random effect patient (to control for individual variation). To explain the vocalization trajectory, three fixed effects were considered: Group (ASD vs. TD), Time (T1 vs. T2 vs. T3), and Face-gazing (yes vs. no). In order to investigate the effect of the interaction Group × Time, an ANOVA was used to compare the additive model without interaction, and the model with interaction. To assess the validity of the model, we also calculated the Akaike information criterion (AIC) and the Bayesian information criterion (BIC).

## Results

General information (means and *SD*) on the rate per minute of vocalizations, babbling and first words in the ASD and TD groups over the three time periods (T1,T2,T3), are reported in Table 4. Between-group differences (results from the Mann–Whitney test) are presented in Table 5, and within-group differences (results from Friedman and Wilcoxon tests), are reported separately for ASD and TD, in Tables 6, 7.

**Table 4:** **Means and standard deviations of rate per minute of Vocalizations, Babbling (Long reduplicated babbling; 2-syllable babbling), and First words in the ASD and TD groups, at T1, T2, and T3**.

	**Mean (Standard Deviation)**					
	**ASD**			**TD**		
	**T1**	**T2**	**T3**	**T1**	**T2**	**T3**
Vocalizations	4.82 (3.10)	2.53 (2.86)	2.73 (1.54)	4.70 (3.50)	6.17 (2.38)	4.54 (2.70)
Long reduplicated babbling	0.00 (0.00)	0.07 (0.14)	0.11 (0.18)	0.00 (0.00)	0.11 (0.30)	0.03 (0.09)
Two-syllable babbling	0.00 (0.00)	0.05 (0.13)	0.48 (0.91)	0.00 (0.00)	0.15 (0.26)	0.78 (1.20)
First words	0.00 (0.00)	0.03 (0.09)	0.00 (0.00)	0.00 (0.00)	0.00 (0.00)	1.40 (1.92)

**Table 5:** Between-group differences are reported using Mann-Whitney Test, level of significance is set at ^*^p < 0.05 and ^**^p < 0.01.

	**Between group comparison (ASD vs TD) Mann-Whitney test**		
	***Z*-values (T1)**	***Z*-values (T2)**	***Z*-values (T3)**
Vocalizations	−0.18	−2.61^**^	−1.02
Long reduplicated babbling	0.00	−0.11	−1.15
Two-syllable babbling	0.00	−0.74	−0.94
First words	0.00	−0.95	−2.18^*^

**Table 6:** **Within-group differences in the ASD group are reported using Friedman Test**.

	**ASD Group (Within group comparison)**			
	**Friedman test**		**Wilcoxon signed ranks test**	
	**Chi-square**	***P*-value**	***Z*-values (T1–T2)**	***Z*-values (T2–T3)**
Vocalizations	6.89	0.03	−2.80^*^	−1.24
Long reduplicated babbling	2.80	0.25	−1.34	−0.41
Two-syllable babbling	11.20	0.004	−1.34	−2.20
First words	2.00	0.37	−1.00	−1.00

Z-values from post-hoc analysis with Wilcoxon signed-rank tests are also reported (significance level is set at ^*^p < 0.017).

**Table 7:** **Within-group differences in the TD group are reported using Friedman Test**.

	**TD Group (Within group comparison)**			
	**Friedman test**		**Wilcoxon signed ranks test**	
	**Chi-square**	***P*-value**	***Z*-values (T1-T2)**	***Z*-values (T2-T3)**
Vocalizations	5.25	0.07	−0.98	−1.72
Long reduplicated babbling	1.00	0.61	−1.00	−0.54
Two-syllable babbling	11.20	0.004	−1.34	−1.69
First words	8.00	0.02	−0.00	−1.83

*Z-values from post-hoc analysis with Wilcoxon signed-rank tests are also reported (significance level is set at p < 0.017)*.

### Vocalizations

**Figure 1:**
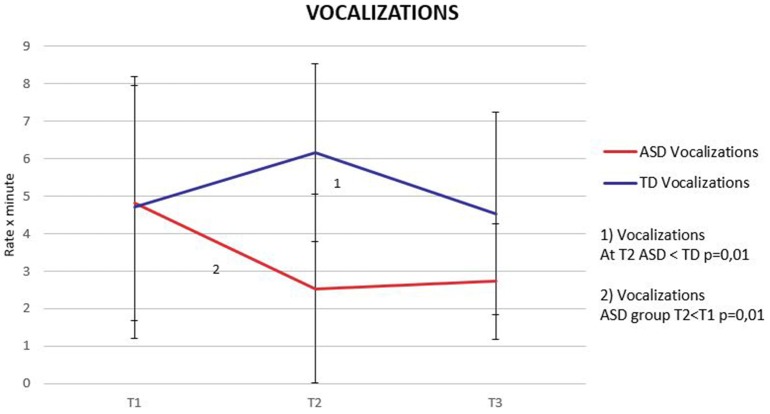
**Vocalization trajectories**. Means and standard deviations (error bars) during the three time periods in the ASD and TD groups.

Using Mann-Whitney non-parametric test, no differences were found between groups in the rate per minute of Vocalizations at T1. To further examine our null finding we calculated an *r* effect size obtained in the following way: *r* = Z/√N. We found a small effect size (*r* = 0.04) suggesting that at this age we do not have evidence of differences between the two groups. At T2, the rate per minute of Vocalizations significantly distinguished the two groups, with the ASD group presenting significantly fewer vocalization acts compared to the TD group (*p* = 0.009) (Figure 1). Within-group analysis, performed using Friedman test, identified the presence of significant differences in the rate of Vocalizations over time (*X*^2^ = 6.89; *p* = 0.03). Therefore, Wilcoxon signed-rank test was applied, evidencing a significant decrease in Vocalizations between T1 and T2 in the ASD group (*p* = 0.005). To explore these changes in vocalization during early development, we also used a generalized mixed model with a random effect “patient” and considered 3 fixed effects: Group (ASD vs. TD), Time (T1 vs. T2 vs. T3), and Gazing to face (yes vs. no). In order to investigate the effect of the interaction Group × Time, an ANOVA was used to compare the additive model without interaction, and the model with interaction. The *X*^2^-test was significant (*X*^2^ = 8.15, df = 2, *p* = 0.017) indicating a better fit of the model with interaction (AIC = 240.68, BIC = 264.98). Thus the interaction model was retained. We found a significant group effect showing more vocalization in TD infants (β = 0.42, *p* = 0.02), and a significant gazing to face effect showing less vocalization when gazing to face (β = −0.49, *p* < 0.001). The interaction term showed a significant increase of vocalization in TD between T1 and T2 (β = 0.85, *p* < 0.001).

### Babbling

**Figure 2:**
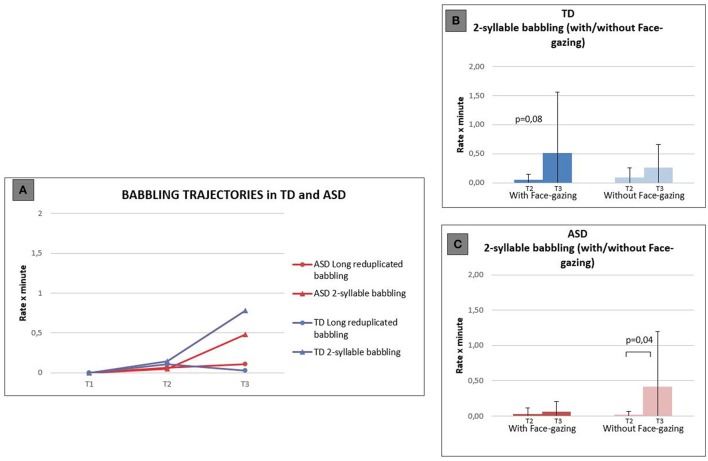
**Babbling trajectories during the three time periods in the ASD and TD groups (graph A)**. In the two windows (graphs **B,C**), differences in the coupling of 2-syllable babbling with and without Face-gazing are illustrated both for the TD group **(B)** and ASD group **(C)**.

The Mann-Whitney test did not show any significant differences in the rates per minute of Babbling behaviors between ASD and TD, at any time period. To further examine our null findings we calculated an *r* effect size obtained this way *r* = Z/√N. Both for Long reduplicated babbling and 2-syllable babbling we found relatively small effect sizes suggesting we have no evidence that the groups differ in their ability to babble: Long reduplicated babbling (T2, *r* = 0.02; T3, *r* = 0.26); Two-syllable babbling (T2, *r* = 0.17; T3, *r* = 0.2). Figure 2 graph **A** illustrates similar babbling trajectories for ASD and TD children from T1 to T3. The qualitative analysis, investigating the association of vocal productions with Face-gazing, was performed with Wilcoxon test (with significance level set at *p* < 0.017 by Bonferroni's correction) and found some trends in within-group differences. Results showed in fact a trend for an increase in 2-syllable babbling not accompanied by Face-gazing in the ASD group between T2 and T3 (*p* = 0.04) (graph **C**), whereas in the TD group, the increase in 2-syllable babbling tended to be associated with Face-gazing (*p* = 0.08) (graph **B**). These differences, however, were not sustained by between-group analyses with the Mann-Whitney test, probably due to the large standard deviations we found and to the small sample size.

### First words

**Figure 3:**
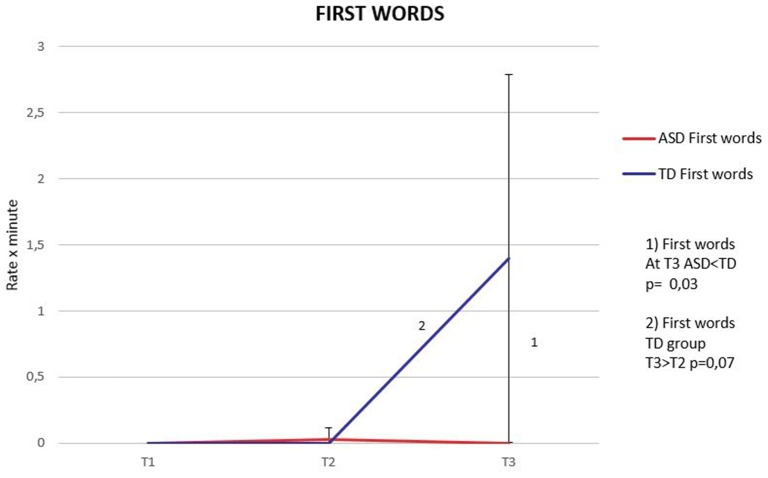
**First word trajectories**. Means and standard deviations (error bars) during the three time periods in the ASD and TD groups.

The Mann–Whitney test showed a significant difference in First words production in favor of TD children at T3 (*p* = 0.03). When we investigated within-group differences, the Friedman test showed a significant difference between time periods (*X*^2^ = 8.00; *p* = 0.02), however, when we conducted *post-hoc* analysis with Wilcoxon signed-rank test and applied Bonferroni correction (significance level set at *p* < 0.017) we found a non-significant trend for increase in First words between T2 and T3 (*p* = 0.07) (Figure 3).

## Discussion

The purpose of this study was to describe pre-linguistic vocal trajectories in infants subsequently diagnosed with ASD in a comprehensive way by examining their quantitative and qualitative aspects, during naturalistic caregiver-child interaction. The identification of prodromal signs (pre-clinical signs that precede the appearance of the full-blown clinical picture of an ASD) is in fact fundamental for the development of preventative interventions, which attempt to ameliorate early presentation and even “prevent” emergence of the full syndrome (Yirmiya and Charman, 2010). In the following paragraphs, the results of the comparison with typically developing peers will be discussed, in reference to our initial hypotheses.

### Similar rates of vocalizations characterize ASD and TD infants at 0–6 months

Our study did not find any significant difference in the rate of Vocalizations between ASD and TD infants during the first 6 months of life. This result is supported by other studies, which analyzed the rate of vocalizations in the first 6 months of life and found similar rates in ASD and TD infants (Ozonoff et al., 2010; Rozga et al., 2011; Apicella et al., 2013). Contrary to our hypothesis, when we explored the presence of Face-gazing coupled with Vocalizations, infants with ASD presented a similar pattern to that of TD infants. Data on gaze behavior during the first months of life in ASD presents great variability across the literature. Indeed there are studies that report that eye looking is present but in decline within the first 2–6 months of life (Jones and Klin, 2013), while others report consistent gaze to the eye region and typical affective behavior in 6 month-olds subsequently diagnosed with ASD (Young et al., 2009). These results seem to suggest that young children that go on to develop autism may show typical behavior in social-vocal patterns during the first 6 months of life. At this age, prodromal signs could be looked for in other areas of development such as motor development, which shows atypicalities starting from the first months of life (Phagava et al., 2008; Bhat et al., 2012) or they might be found investigating the acoustic characteristics of infant's cries (Sheinkopf et al., 2012; Esposito et al., 2013). Nevertheless, we have to consider that we did not include “grunts” in our Vocalizations category. “Grunts” emerge early in infants and have been found to be specifically predictive of early referential words in typical development (McCune et al., 1996; McCune and Vihman, 2001); thus, in the future their analysis could improve the detection of differences in early vocalizations between ASD and TD.

### During the 6–12 months period, vocal trajectories of ASD and TD infants diverge

While TD infants increased their production of Vocalizations during the 6–12 months period, conversely, infants in the ASD group produced significantly fewer Vocalizations (Figure 1). An atypical vocalization trajectory during the first year of life was also described by Ozonoff et al. (2010) in siblings who later developed an ASD, who at the age of 6 months were not significantly different in their vocalizations or smiles compared to TD, while at the age of 12 months a reduction in the frequency of vocalizations occurred.

In accordance with other studies, it is possible that these differences might be related to changes in reciprocal and intentional social communicative behaviors which appear to increase during the second semester in typical development, whereas in ASD there is a declining slope that culminates in detectable differences by the first birthday (Zwaigenbaum et al., 2005; Winder et al., 2013; Swain et al., 2015). Similarly, Goldberg et al. (2008) have reported that often parents refer, with a good level of accuracy, their child's regression in the domain of early vocalizations. This atypical pattern of Vocalizations suggests that the 6–12 months period could be critical for the detection of prodromal signs of ASD. It is noteworthy that this same period of life appears to be critical for the development of language (Kuhl, 2004) and of cerebral voice processing (Grossmann et al., 2010).

### Possible differences in gaze behavior associated with 2-syllable babbling distinguish ASD and TD infants

The amount of Babbling, contrary to our hypothesis, did not distinguish the two groups. Indeed, we found comparable low levels of Long reduplicated babbling during the 6–18 months period. This result is quite unexpected as it is in contrast with other studies with larger samples which have identified lower rates of canonical babbling in infants with ASD by 12 months (Paul et al., 2011; Patten et al., 2014). It is possible that our result could have been affected by our decision to include simple early babbling (e.g., /ba/) in the Vocalizations category; while this may have contributed to the detection of the different pattern in vocalizations at this age, it may have taken information away from our babbling data, which are non-significant, against prediction. Moreover, as Long reduplicated babbling was not coded much, for either group, it is possible that this result might also have been driven by a floor effect. The small *n* of our samples and intragroup variability may also have influenced our results on 2-syllable babbling, for which we found a similar increase in both groups during the 6–18 months period. However, it is notable that in infants with ASD this increase in 2-syllable babbling does not generally appear accompanied by face-directed babbling while, on the contrary, in TD there is an increase in babbling associated with face-gazing (Figure 2, graphs **B,C**). Nevertheless, between-group analyses did not show significant differences between the two groups, probably because of the large variability in the TD group and of the small sample size. Thus, we were not able to clarify if this effect is driven by the higher frequency of vocalizations associated with Face-gazing in TD children or by the higher frequency of Vocalizations without Face-gazing in ASD. Our finding is similar to that of Winder et al. (2013) who found fewer communicative non-word vocalizations, that is vocal utterances accompanied by eye contact or gesture in infants later diagnosed with ASD. We could hypothesize that infants with ASD have a basic capacity for speech discrimination that is matched across groups, but 2-syllable forms occur without the additional social repertoire in ASD, possibly as a sign or consequence of emerging difficulties in social and intentional behaviors. It could be hypothesized that in ASD solitary babbling is more prolonged than in typical development, as opposed to interactive babbling which is less frequent. Konopczinsky (2010) describes how these two kinds of babbling have different developmental functions: During solitary babbling children could be exploring sensorial variations, while during interactive babbling infants could be taking part in a conversation with a social partner and developing more mature language skills. Hence, we could speculate that at this age, infants later diagnosed with ASD are focused more on sensory seeking and are less engaged in interacting with their social partners.

### First words: a delayed milestone in ASD

In the ASD group the increase in Babbling does not culminate in the production of First words, as in the TD group (Figure 3). During the 12–18 months period in fact, we found a significant difference in the rate of First words, which were produced with higher frequency in the TD group. These results seem to indicate a delay in the achievement of this verbal milestone in the ASD group, as reported accurately by parents (Mitchell et al., 2006; Goldberg et al., 2008) whose first concern is often represented by the delay of first words. We can speculate about the nature of this delay considering that the increase in 2-syllable babbling in infants with ASD was characterized mainly by an increase of babbling behavior not associated with gaze behavior directed toward the social partner. Given that language develops in a social context, trying to imagine what the consequence of this behavior might be at the level of caregiver-child interaction, we could hypothesize that if infants don't direct their babbling to their social partners, this might make it harder for caregivers to attribute a social meaning to their infant's vocal productions and to reply suggesting words according to the context. Thus, compared to their typically developing peers, children with ASD might be engaged in fewer vocal exchanges with their social partners, and could be less exposed to language which is attuned to their feelings or interests, making it more difficult for them to learn language.

## Conclusions

Summarizing our results, a distinctive pattern of early pre-linguistic vocal behavior emerges in infants subsequently diagnosed with ASD. After an initial regular display of Vocalizations, at 6–12 months there is a decrease in Vocalizations in infants with ASD, as opposed to the increase found in TD infants. At 6–18 months Babbling emerges and increases progressively both in ASD and TD infants. However, at 12–18 months 2-syllable babbling appears to be less likely to be accompanied by Face-gazing in toddlers with ASD. On the contrary, at this age, in TD this babbling pattern is mainly associated with Face-gazing. After the first birthday, the delay in first words characterizes toddlers with ASD distinguishing them from their typically developing peers.

These findings could contribute to the open debate on continuity or discontinuity of vocalization with early speech (babbling/first words) (Vihman et al., 1985; Karousou and López Ornat, 2013). In our study the decrease in vocalizations in infants with ASD does not seem to have a negative effect on the emergence and development of babbling suggesting a discontinuity between early vocalizations and babbling specific to ASD. On the other hand, typical development seems characterized by a certain continuity of vocalizations with babbling and first words. Our findings suggest that the continuity between vocalizations, babbling and the emergence of first words is driven by the prevalence of socially-oriented babbling, which is lacking in ASD.

Our results could also have implications for early screening: We propose that in the future, screening tools should investigate atypical vocal patterns, and in particular the decrease in vocalization during the second half of the first year of life and the lack of coordinated eye gazing. Some instruments for early screening have already included useful items on vocal productions. For example, the First Year Inventory (FYI, Reznick et al., 2007), which is a parent-report measure designed to identify 12-month-old infants at risk for ASD, includes an item which asks parents if their baby babbles by putting sounds together, such as “ba-ba,” “ga-ga-ga,” or “ba-dee.” Moreover, vocalizations, as a part of the FYI construct of Imitation, have been found to be the greatest distinguishing factor between ASD cases and children with other outcomes (Rowberry et al., 2015). This kind of instrument appears very promising because it could easily be used by the child's doctor to collect information from parents at well-child visits and it could be fundamental for identifying children that require further testing with more specific clinical instruments, which are designed to assess the risk of autism in infants and toddlers (e.g., Autism Observation Scale for Infants–AOSI Bryson et al., 2008), ADOS-2 Toddler Module by Lord et al. (2012). In these standardized assessments, specific attention is also dedicated to the frequency of spontaneous socially-directed vocalizations, indicating the importance of assessing early communication abilities also in infants.

The results from this study could also have implications for early intervention. Early investigation of both the frequency and quality of vocal behaviors could in fact facilitate the identification of possible divergences from typical development and help us to address them promptly through early preemptive intervention. Leezenbaum et al. (2013) have pointed out that early delays or atypicalities in vocal development might influence parental responses as well, (e.g., providing fewer opportunities for parents to verbally label a gesture referent), and this could in turn alter the input that these infants receive, with cascading effects on the subsequent development of language. Thus, preemptive intervention could aim to inform and help parents interact in ways that increase their infant's attentiveness and spontaneous communicative acts. However, more research still needs to be done to better understand which kinds of strategies work best for promoting language and communication development in infants at risk for autism. The first randomized early intervention trial for infants at high risk for autism was conducted by Green et al. (2015). In their parent-mediated intervention for infants aged 9–14 months old they found that an increase in parental non-directiveness was associated with an increase in infants' attentional flexibility and a reduction in atypical behaviors on the AOSI, but they did not find that the intervention had any effect on developmental language measures or on responsiveness to language sounds measured with auditory ERP. The authors suggest this result could indicate an atypical pathway for language learning in at risk-infants, which is not fostered by parent non-directiveness as in typical development. Thus it is fundamental to investigate how language may develop differently in infants who develop ASD, and how other abilities may influence language learning. Interventions working on improving functioning in other areas of development, such as gross-motor and oral-motor abilities or imitation skills may indeed foster language development as well.

In conclusion, our study offers some preliminary findings about pre-linguistic vocal development in infants later diagnosed with an ASD. However, it also has some important limitations. First of all, our small sample size enables us to consider the results as only preliminary. Secondly, our ASD group is characterized by low-functioning children, so it would have been useful to include also a group of children with developmental delay, to verify whether our results are specific to autism or rather the consequence of a global delay. Although our sample is not representative of the ASD population in general, it is in line with data from the literature which states that approximately two thirds of individuals with ASD have co-occurring intellectual disability (Dykens and Lense, 2011). Another crucial aspect, which should be considered carefully when designing studies on highly variable material such as preverbal vocalizations is related to how the coding scheme is set up (which behaviors are investigated, and how they are coded). In our study, for instance, some of the decisions at the coding stage (e.g., the exclusion of “grunts” and the inclusion of early babbling in the Vocalizations category or the exclusion of “jargon” from the First words category) may have affected the results. Furthermore, while retrospective studies using home videos have proved to have high ecological validity, they have several limitations related to the non-homogeneous quality of the material collected by families and to the selection of specific moments, not necessarily representative of the infant's general behavior (Costanzo et al., 2015). In the future, some of these limitations could be overcome using prospective designs, whereby the researcher has greater control of the experimental setting and conditions.

## Author contributions

NC, DW, VC, AD, ML, EP, DC, FA, SC, and FM: Participated in the design, execution, and analysis of the paper by NC and colleagues, entitled “Pre-linguistic vocal trajectories at 6–18 months of age as early markers of autism,” and declare they have seen and approved the final version and that it has neither been published nor submitted elsewhere.

## Funding

SC was supported by the Ministry of Health, Italy and by Tuscany Region with the grant “GR-2010-2317873,” and by Bando FAS Salute Sviluppo Toscana -ARIANNA Project-. This work was also supported by a grant from the IRCCS Stella Maris Foundation (Ricerca Corrente, and the “5 × 1000” voluntary contributions, Italian Ministry of Health).

### Conflict of interest statement

The authors declare that the research was conducted in the absence of any commercial or financial relationships that could be construed as a potential conflict of interest.
